# Evaluation of the GPS Precise Orbit and Clock Corrections from MADOCA Real-Time Products

**DOI:** 10.3390/s19112580

**Published:** 2019-06-06

**Authors:** Shaocheng Zhang, Shikang Du, Wei Li, Guangxing Wang

**Affiliations:** School of Geography and Information Engineering, China University of Geosciences, Wuhan 430074, China; zsc@cug.edu.cn (S.Z.); dsk@cug.edu.cn (S.D.); wanggx@cug.edu.cn (G.W.)

**Keywords:** QZSS satellite, MADOCA, orbit and clock errors, precise point positioning

## Abstract

The Japanese Quasi-Zenith Satellite System (QZSS) is a regional navigation satellite system covering the entire Asia-Oceania region. Except for the standard satellite navigation signals, QZSS satellites also broadcast L6E augmentation signals with real time GNSS precise orbit every 30 s and clock messages every 1 s, which is very important and necessary for Real-Time precise point positioning (RTPPP) applications. In this paper, the MADOCA real-time services derived from L6E augmentation signals were evaluated for both accuracy and availability compared with IGS final products. To avoid the datum difference of GPS orbit between MADOCA real-time and IGS final products, the 7-parameters Helmert transformation was firstly used in this paper, and then the orbit was evaluated on radial, along, and cross-track directions. On the clock evaluation, the mean satellites clock errors were taken as reference clock error, and then the standard deviation (STD) was calculated for each satellite. Furthermore, the signal in space range errors (SISRE) were also summarized to evaluate the ranging-measurement accuracy. Seven-day evaluation results show that satellite orbit, clock errors, and the final SISRE errors range as being 1.8–3.9 cm, 0.04–0.15 ns (1.2–4.5 cm), and 5–10 cm, respectively. For the one-year long-term evaluation, daily SISRE errors in 2018 show consistent performance with that of seven days. Furthermore, the open source software RTKLIB was used to evaluate the kinematic PPP performance based on the MADOCA real-time products, and it shows that the daily positioning accuracy of the 20 globally distributed IGS stations can reach 4.9, 4.2, 11.7, and 12.1 cm in the east, north, up, and 3D directions, respectively. Hence, it is concluded that the current MADOCA real-time ephemeris products can provide orbit and clock products with SISRE on centimeters level with high interval, which could meet the demands of the RTPPP solution and serve real-time users who can access the MADOCA real-time products via L6E signal or internet.

## 1. Introduction

Quasi-Zenith Satellite System (QZSS) is a regional satellite navigation system covering the entire Asia-Oceania regions. The first Quasi-Zenith Satellite (QZS-1, nicknamed ‘Michibiki’) was launched on September 11, 2010 and operated by the Japan Aerospace Exploration Agency (JAXA) [1]. The Cabinet Office, Government of Japan (CAO) took control of QZS-1 since 28 February 2017 [2], then developed and launched additional three satellites in 2017. On 1 November 2018, CAO claimed that QZSS started to provide services officially [3], which is currently composed of three quasi-zenith orbit (QZO) satellites and one geostationary orbit (GEO) satellite and will be extended to a seven-satellite system in 2023 [4].

The QZO satellite can provide continuous signal coverage at a high elevation angle and improve the visibility of QZSS satellites over Japan even if there are many urban canyons and mountainous areas [5]. The QZSS satellites could act as complementary navigation satellites by broadcasting exactly the same navigation signals as GPS satellites [6] and also extend to satellite-based augmentation system (SBAS) by transmitting high-accuracy GNSS satellite orbit and clock correction via satellite linkage for real-time precise point positioning (RTPPP) applications [7].

On RTPPP applications, the accuracy of satellite orbit and clock products is a prerequisite because they are introduced to the PPP observation equations as known parameters [8], thus the errors on the orbit/clock would be propagated to the positioning results and affect the positioning accuracy. Since the QZS-1 was successfully launched, evaluation researches had been started for users in the Asia-Oceania area [7,9,10]. However, in the early period, the QZSS real-time orbit and clock products are not generated based on global distributed ground stations, and the positioning performance shows obvious spatial difference [9]. Furthermore, the update rate of 180 s of QZSS real-time products in the early period is not frequent enough; the interpolation on orbit and clock products would thus introduce uncertainty to the real time positioning results [11,12].

**Figure 1:**
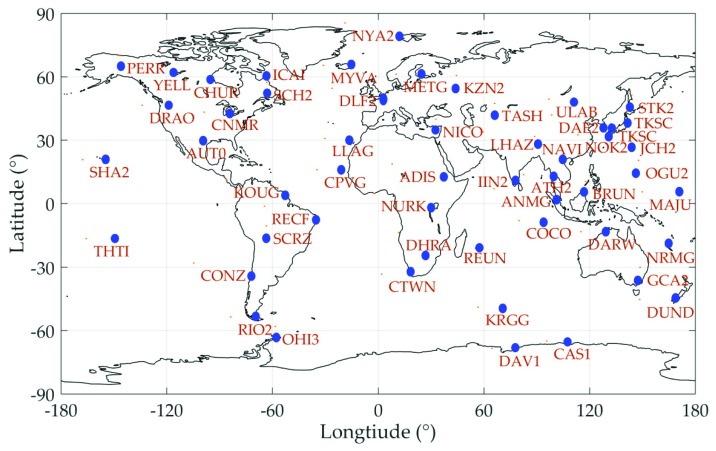
The ground stations for the MADOCA real-time ephemeris generation.

After the successful transfer of QZS-1 control to CAO, the trial service of Centimeter Level Augmentation Service (CLAS), called CLAS Experiment (CLAS-E), started to provide centimeter level augmentation information on March 28, 2017 [13]. These satellite-based augmentation messages are mainly high rate precise satellite orbit and clock products, aiming to meet the requirement of the real time PPP application for users who can receive L6E messages either from QZSS satellite directly or via internet [14]. The augmentation message of CLAS-E is using MADOCA products generated by the software named Multi-GNSS Advanced Demonstration tool for Orbit and Clock Analysis (MADOCA) developed by JAXA. The current MADOCA real-time orbit and clock products are calculated based on real-time data streams from 53 CORS stations distributed as in Figure 1 [15].

This research aimed to perform a comprehensive evaluation of the MADOCA real-time augmentation service from the aspect of GPS satellite products availability, accuracy, and positioning performance. Data latency evaluation is ignored as it highly dependent on the user’s hardware or internet performance. The paper is organized as follows: This section is a brief review of the QZSS system including its development and augmentation service; and then, the data sets and evaluation strategy for the MADOCA real-time orbit and clock products is introduced in Section 2; in Section 3, the products availability is evaluated; and accuracy of the MADOCA real-time products are analyzed by comparing these with the IGS final products in Section 4; Section 5 discusses the calculation method of the SISRE metrics and obtains the corresponding statistical results; in Section 6, the simulated RTPPP with over 20 IGS station data was applied to evaluate the real-time positioning performance in different areas; finally, conclusions are summarized in Section 7.

## 2. Data Sets and Evaluation Strategy

Currently, the MADOCA real-time products could be obtained by RTCM SSR via internet, satellite, as well as RINEX SP3 format [14]. Considering the flexible accessibility and stability of products in the short- and long-term, the recorded MADOCA real-time products utilized in this paper were accessed from the JAXA website in SP3 format [16], which provides non-redundant, time-sorted real-time data records for GPS, GLONASS, and QZS-1 satellites including orbit, clock, and user range accuracy (URA) messages at 30-s intervals. The IGS final products from IGS data center of Wuhan University (www.igs.gnsswhu.cn) were taken as reference products for the products availability and accuracy evaluations.

On the products availability evaluation, the integrality of the MADOCA real-time orbit and clock messages was accessed by comparing with IGS orbit and clock records epoch by epoch with over three month statistics, from 1 November 2018 to 31 January 2019.

Moreover, on the products accuracy evaluation, the 7-parameters Helmert transformation was firstly conducted with daily products to unify the coordinate datum on the orbit evaluation, and the clock products were evaluated with the standard deviations by taking the mean satellite clock as reference. To avoid interpolation errors, the accuracy comparison intervals were taken exactly the same as IGS products, which are 15 min for orbits and 30 s for clocks. And the statistics of the SISRE were also used to evaluate the MADOCA products accuracy on the ranging measurement.

Over 20 globally distributed IGS stations were used to simulate the real time PPP performance with the open source RTKLIB package. The selected IGS stations are chosen evenly distributed but avoiding the MADOCA network stations, and the data processing interval is taken as 30 s to avoid the infection of interpolation errors.

## 3. Products Availability

**Figure 2:**
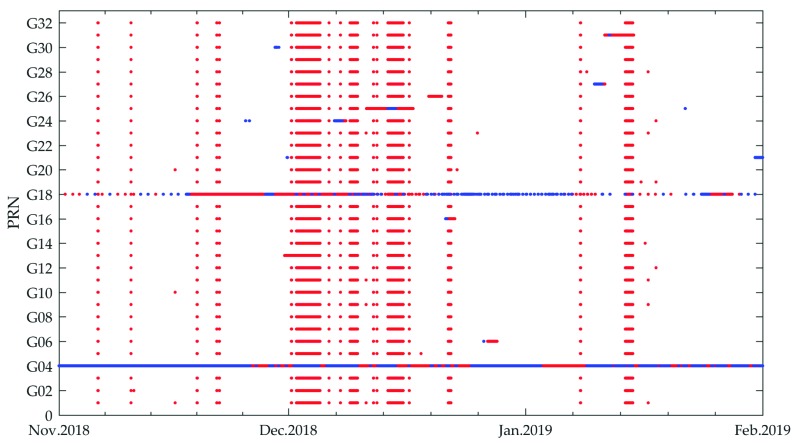
Unavailability of MADOCA real-time products for GPS satellites from 1 November 2018 to 31 January 2019.

To investigate the availability of MADOCA products, the MADOCA recorded real-time products and the IGS final products were collected from 1 November 2018 to 31 January 2019. The three-month statistic results are presented in Figure 2. The red dots represent the satellite unavailability only in MADOCA real-time products, and the blue dots denote the satellite unavailability in both MADOCA and IGS final products.

It should be noted that in early December 2018, there was a serious absence of MADOCA real-time products which was officially explained by JAXA as an unplanned stop providing MADOCA real-time products [17]. Furthermore, the G04 was always on unhealthy status in the period because no MADOCA and IGS final products were available for this satellite at all. In addition, the availability of the G18 was also not good, given the low-quality MADOCA real-time products. In the following availability and accuracy assessment, these two satellites were excluded from the statistics. Statistics show that the epoch availability of MADOCA real-time products during the three months was about 92.6% and the available number of satellites at almost all of available epochs can reach 30, which makes it possible to determine a high quality positioning geometry in real-time PPP applications.

## 4. Orbit and Clock Evaluation

### 4.1. Unification Coordinate Datum

Considering the geodetic datum of satellite orbit difference between MADOCA real-time and IGS final products [18], the Helmert transformation is used to ensure the consistency of the reference frame. This 7-parameter transformation model can be expressed as follows:

(1) $$\left[\begin{array}{c} X_{T}\\ Y_{T}\\ Z_{T} \end{array}\right]=M*\left[\begin{array}{ccc} 1 & -R_{Z} & +R_{Y}\\ +R_{Z} & 1 & -R_{X}\\ -R_{Y} & +R_{X} & 1 \end{array}\right]*\left[\begin{array}{c} X_{S}\\ Y_{S}\\ Z_{S} \end{array}\right]+\left[\begin{array}{c} dX\\ dY\\ dZ \end{array}\right],$$

where ${\left(\begin{array}{ccc} X_{S} & Y_{S} & Z_{S} \end{array}\right)}^{T}$ and ${\left(\begin{array}{ccc} X_{T} & Y_{T} & Z_{T} \end{array}\right)}^{T}$ are the coordinates of the point at the same epoch in the source geocentric coordinate reference system (i.e., JGD) and the target geocentric coordinate reference system (i.e., ITRF), respectively. In addition, ${\left(\begin{array}{ccc} dX & dY & dZ \end{array}\right)}^{T}$ denotes the translation vector, $\begin{array}{c} R_{X} \end{array}$, $\begin{array}{c} R_{Y} \end{array}$ and $\begin{array}{c} R_{Z} \end{array}$ are the three rotation parameters, and M is the scale correction parameter, which is used to the scale correction in the position vector in the source coordinate reference system.

To solve these seven parameters, 3D coordinate data of 96 epochs in one day as well as the least-squares algorithm are applied to obtain the most probable value of seven parameters for each satellite. Then, transformation between the two coordinate references can be achieved by Equation (1).

### 4.2. Clock Error Statistic

Similar to the evaluation process of the orbit error, clock-offset products comparison between different Analysis Centers (ACs) typically cannot be directly made because of differences in the choice of the GPS system timescales which influence all satellites of a constellation in the same way and result in a constant systematic deviation [19]. In order to eliminate the deviation, an ensemble clock error should be counted and applied to the clock errors of each satellite. As such, the method of selecting one satellite as a reference (so-called single-satellite method) is typically adopted by most literature [20,21]. This method requires a reference satellite which has the highest clock-offset precision, otherwise the evaluation results of all the other satellites will be larger than their real values, but such a reference is usually not easy to determine. Therefore, Yao et al. proposed the so-called all-satellite reference method [22] which selects all satellites in the same epoch to participate in the calculation of the system deviation. The solution formula of this method is adopted in this research and it can be expressed as:

(2) $$∆t_{e}^{i}=T_{e}^{i}-t_{e}^{i}-\mu_{e},$$

where

(3) $$\mu_{e}=\sum_{i}^{n}\frac{\left(T_{e}^{i}-t_{e}^{i}\right)}{n}$$

denotes the average of precise-minus-MADOCA clock values of all satellites (i.e., timescale difference), which is calculated epoch-wisely. The variation of average does not affect the positioning results, since the average is the same for all satellites and would be absorbed by the estimable receiver-clock offsets of PPP users at each epoch. The number of satellites is n. $T_{e}^{i}$ and $t_{e}^{i}$ are the precise clock bias and MADOCA clock bias, respectively. Subsequently, the individual clock systematic deviations are corrected by this ensemble average. It is worth noting that in this comparison the gross error caused by the clock outliers or non-existence of the clock-offset values in the raw data is eliminated.

### 4.3. Orbit/clock Statistic Accuracy Results

**Figure 3:**
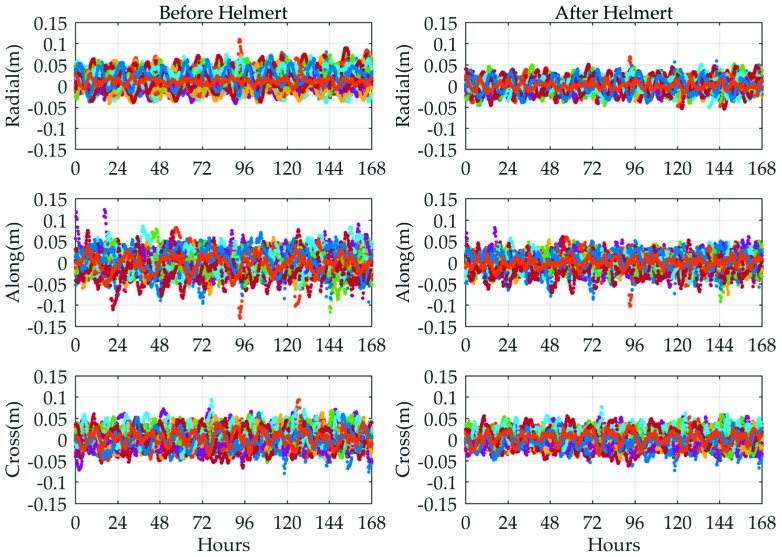
Time series of the MADOCA real-time GPS satellite orbit errors in radial and along/cross-track directions from 11 November 2018 to 17 November 2018.

**Figure 4:**
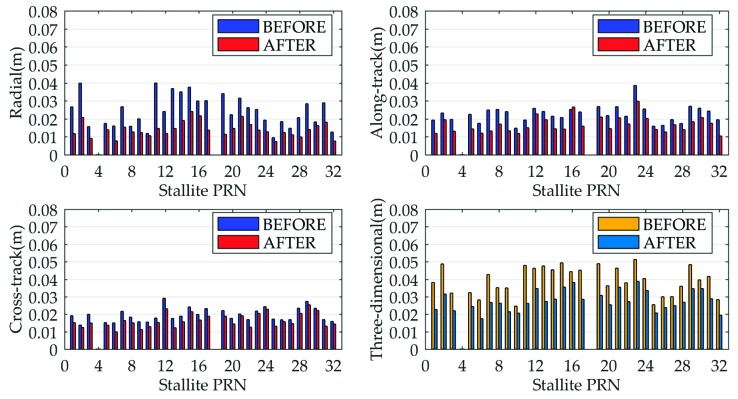
Seven-day average-RMS errors of all available GPS satellites in radial, along/cross-track, and three-dimensional (3D) directions.

Based on the concepts and algorithms described above, the short-term orbit difference between the MADOCA recorded real-time and IGS final products before and after Helmert transformation was determined from 11 November 2018 to 17 November 2018. The corresponding comparison results are collated in Figure 3. It shows the orbit comparison of all available GPS satellites between the MADOCA and IGS final products in radial and along/cross-track components before and after Helmert transformation. The different color points represent different GPS satellites. To quantify the orbit accuracy of each satellite in three directions, the seven-day RMS for each direction and three-dimensional (3D) RMS values are represented in Figure 4.

As shown in Figure 3, the orbit errors in radial direction for all GPS satellites show better performance compared to the other two directions; the accuracy differences among the three components, however, are not significant. It can be seen from Figure 4 that the orbit errors of all available satellites in 3D component are within 5 cm, which basically meets the accuracy goals of MADOCA real-time products.

In addition, special attention needs to be paid to the fact that all satellite orbit errors are improved after conducting the 7-parameters Helmert transformation, particularly in the radial direction, and which can be further verified from subsequent SISRE (orb) value plots. After the coordinate reference is unified, the average-RMS values of the MADOCA real-time orbits are roughly less than 2 cm in the three components and below 4 cm in the 3D direction.

**Figure 5:**
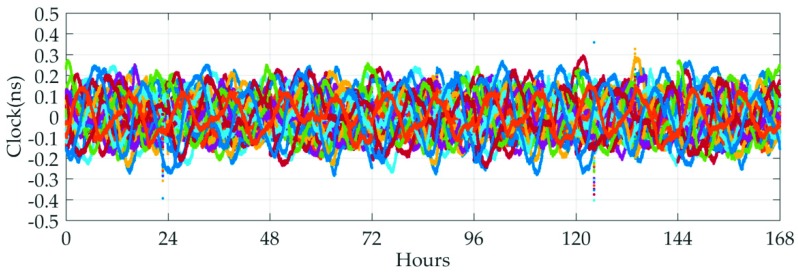
Time series of the MADOCA GPS satellite clock errors from 11 November 2018 to 17 November 2018.

**Figure 6:**
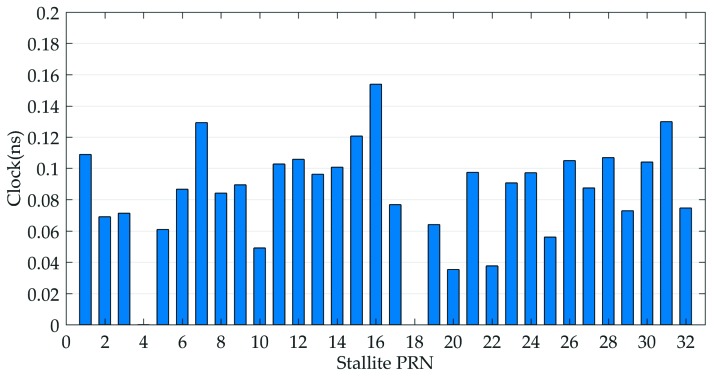
Seven-day average-STD clock errors of all available GPS satellites.

Afterwards, the IGS final clock products are utilized as reference to assess the quality of the GPS real-time clocks provided by MADOCA products. The STD values, rather than RMS, are taken as the evaluation indexes because the mean biases of GPS clock offsets can be absorbed by ambiguity parameters and will not affect the positioning results [23,24]. Figure 5 shows the clock errors of all available GPS with respect to IGS products, and the average-STD of clock differences are given in Figure 6.

As show in Figure 5 and Figure 6, the various generations of GPS satellites exhibit the better consistency in clock accuracy, which will ultimately be reflected in the respective SISRE budgets. Again, one-day periodical variations are visible for almost all GPS satellites. For each GPS satellite, the averaged-STD value is typically better than 0.16 ns and most of them are at the 0.1 ns level, which confirms that the satellite clocks show a good short-term stability and are capable of achieving the accuracy at cm level in real-time applications.

## 5. SISRE Computation

The SISRE is a comprehensive measure of the fidelity of the MADOCA products, reflecting the combined effects of satellite orbit and clock errors projection in the line of sight. The SISRE’s contributions and statistical characterization are at the same time of crucial significance for the development of advanced-receiver autonomous-integrity monitoring (ARAIM) algorithms.

The instantaneous SISRE calculated by an arbitrary set of orbit errors including radial (R), along-track (A), and cross-track (C) varies with the orientation of the line-of-sight vector and the receiver locations [25]. As such, the concept of global averaging is adopted for SISRE calculation in this paper where “the global average SISRE” means the root-mean-square SISRE across the points within the satellite’s coverage. The GPS Standard Positioning Service performance standard [26] provides two methods for computing the global average SISRE for a satellite at a specific instant epoch. One of them is Brute Force RMS by which the global average SISRE can be computed numerically from instantaneous SISREs, but the amount of calculation is very large; and the other is the Piecewise RMS that computes the global average SISRE by the satellite orbit errors and the total timing error. In this paper we use the latter method.

Accordingly, the following two SISRE metrics are considered in our study, orbit-only contribution to SISRE can be described as:

(4) $$\mathrm{SISRE}\left(\mathrm{orb}\right)=\sqrt{w_{R}^{2}*R^{2}+w_{A,C}^{2}*\left(A^{2}+C^{2}\right)},$$

here, *R*, *A*, *C* denote, respectively, the radial orbit error, along-track orbit error, and cross-track orbit error while $w_{R}$ and $w_{A,C}$ are weight factors for statistical contribution of radial and along/cross-track errors to the line-of-sight ranging error which depend on the altitude of the GNSS satellite [19]. For GPS, $w_{R}$ = 0.98 and $w_{A,C}$ = 0.141.

Analogous to (4), the formula for the global average rms SISRE that combines orbit and clock errors is:

(5) $$\mathrm{SISRE}=\sqrt{{\left(w_{R}R-cT\right)}^{2}+w_{A,C}^{2}*\left(A^{2}+C^{2}\right)},$$

where, *c* and *T* are the speed of light and total timing error, respectively. It can be seen from the equation above that the radial orbit error and the clock error play a vital role in the calculation process of SISRE, and there is a significant negative correlation between them. The SISRE values obtained from Equation (5) can reasonably evaluate the average ranging error of the entire surface of the earth. It is noteworthy that the satellite orbit errors in radial, along-track, and cross-track directions are needed in the SISRE budget, and the performance of the satellite orbit in the radial direction plays a significant role during positioning and navigation, yet the orbit coordinate values provided in these two products are represented in the ECEF frame. Before conducting the evaluation and calculating the SISRE values, therefore, we convert the ECEF frame to RAC frame. The relevant conversion model is assumed to be known and thus will not be discussed. Interested readers are recommended to refer to Hadas and Bosy [8], Mohamed and Salim [27] and Teunissen and Montenbruck [28].

**Figure 7:**
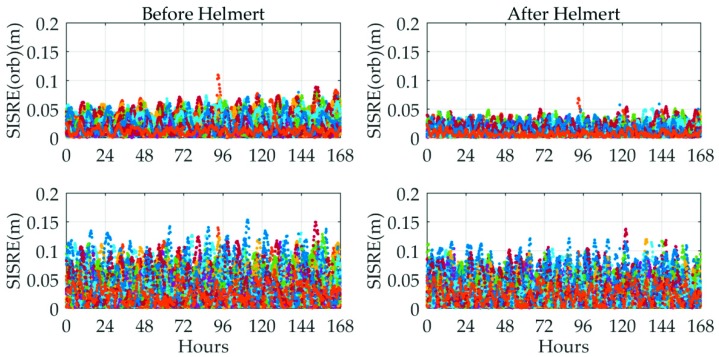
Seven-day signal-in-space range errors of MADOCA real-time products for all available GPS satellites relative to IGS final products from 11 November 2018 to 17 November 2018.

For the purpose of a comprehensive perspective, the orbit-error-only SISRE values and the SISRE values of all available satellites were calculated and the corresponding results are summarized in Figure 7. There are two key messages revealed from this figure. One is that the orbit-error-only SISRE values of all satellites are improved by about 2 to 3 cm after Helmert transformation. The other is that the clock errors contribute an immoderate part of the SISRE budget of almost all GPS satellites. However, the SISRE values are still slightly improved after conducting Helmert transformation. Hence, in the subsequent long-term MADOCA real-time product evaluation, the statistics will be based on the orbit messages after Helmert transformation. As for the SISRE performance, the orbit-error-only SISRE of each satellite achieves a level of 5 cm and the SISRE of each satellite is roughly 10 cm. These results indicate that the MADOCA real-time products achieve JAXA’s goal for real-time orbit and clock accuracy and can meet the centimeter accuracy demands of RTPPP users.

**Figure 8:**
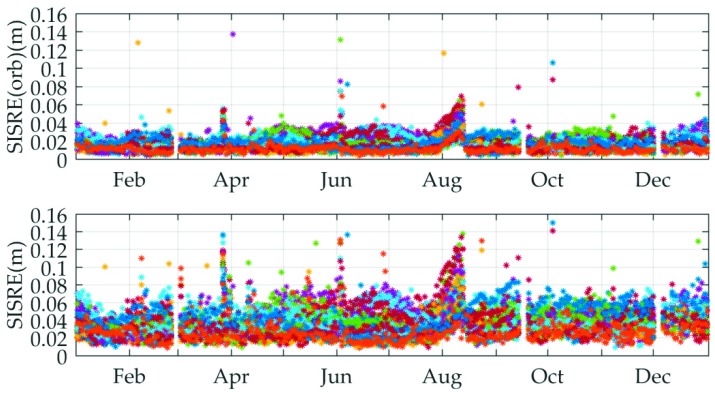
Daily RMS of orbit-error-only SISRE and SISRE of MADOCA real-time products for all available GPS satellites relative to IGS final products from 1 January 2018 to 31 December 2018.

In order to reveal the long-term characteristic of the quality of MADOCA real-time products further, the daily RMS of orbit-error-only SISRE and SISRE values are calculated from the whole year 2018 for all the available GPS satellites. The time series of daily RMS of SISRE are shown in Figure 8.

In Figure 8, we can see that the orbit-error-only SISRE values demonstrate a consistency with short-term values and can basically maintain good stability over the whole year comparison except for a few jumps. Due to the variation in the accuracy of the clock, SISRE values of all available satellites experience relatively large fluctuations, yet most of them are still less than 10 cm. In addition, it is worth mentioning that the reason for the interruption of the graphics in the figure is that the MADOCA raw data is defective or does not exist during these time periods. As such, the raw data in these time periods is considered to be unavailable.

## 6. PPP Validation

**Figure 9:**
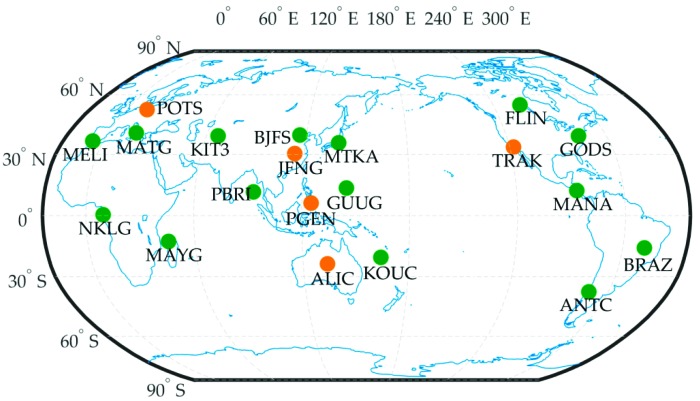
The distribution of 20 IGS stations for PPP validation.

To further verify the quality of current MADOCA real-time products from a practical perspective, PPP experiment was performed using the RTKLIB software developed by Dr. Takasu [29]. GPS observation data of 20 globally distributed IGS stations on 11 November 2018 (DOY 315) were collected for testing with a sampling rate of 30 s. The geographical location of the selected stations for this PPP experiment is shown in Figure 9. In the data processing, the kinematic PPP algorithm provided in the RTKLIB software was applied to process GPS dual-frequency ionospheric-free combination data. To simulate real-time kinematic processing, the forward Kalman filter method was used for PPP validation. The station coordinates provided by the IGS weekly SINEX file (i.e., igs18Pweek.snx) were used as the reference in the positioning error calculations except for the PGEN and MAYG stations. Since there were no precise coordinate messages for these two stations in the IGS SINEX file for the testing periods, the reference coordinates of PGEN and MAYG stations were derived from post-processing of static PPP using IGS final products.

**Figure 10:**
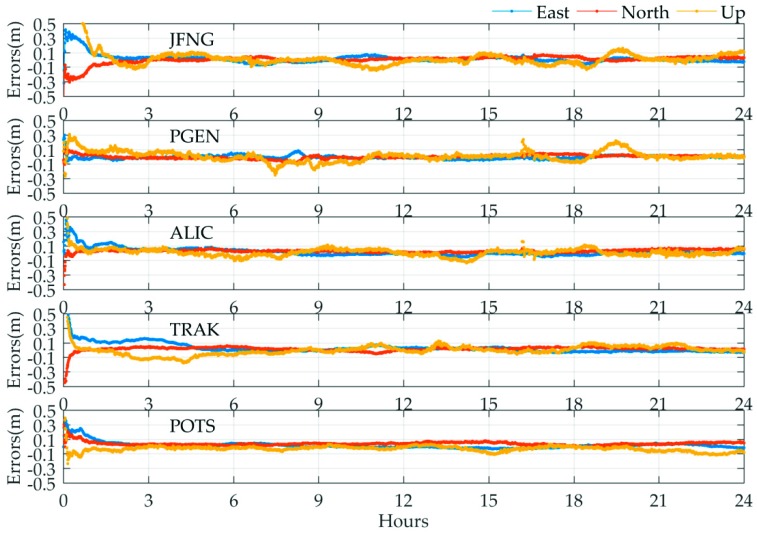
An example of time series of PPP positioning errors on 11 November 2018 for the five IGS stations using the MADOCA real-time product.

Five of the 20 stations were selected to present the accuracy of positioning errors. Their geographical locations have been marked by orange dots in Figure 9. Figure 10 shows the positioning error of the five stations in which the east, north, and up components are denoted by blue, red, and yellow lines, respectively. As seen from Figure 10, the positioning errors of the five stations vary within ±10 cm in the horizontal components and are stable after the convergence/initialization period. For the up component, the positioning errors exhibit more remarkable fluctuation compared to the horizontal components that can even reach up to 15 cm for some epochs, especially a few jumps at the PGEN station. This phenomenon may be explained by the lower accuracy of the clock products in the corresponding epochs.

**Figure 11:**
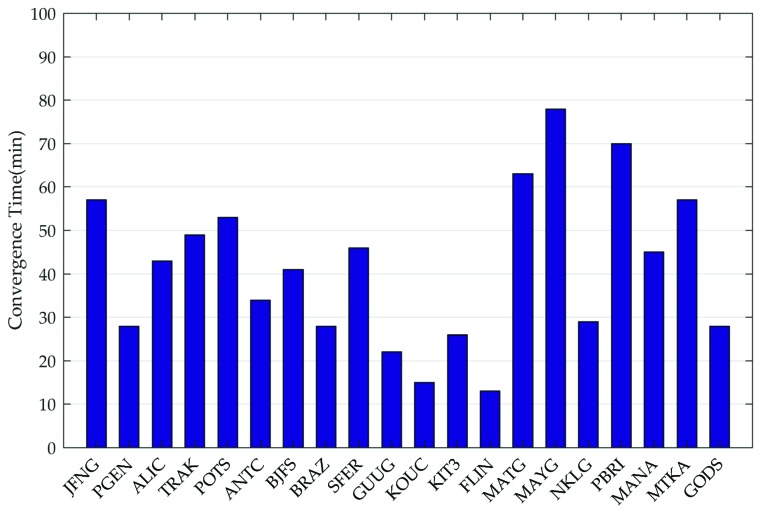
The convergence time for the 20 global distributed IGS stations.

Moreover, the statistics of convergence time for all 20 stations are summarized in Figure 11. We consider the positioning results to be convergent when both horizontal and vertical positioning errors are less than 15 cm over consecutive 20 epochs. From Figure 11, the convergence time of all stations is within 80 min, and the average convergence time is 40 min.

**Figure 12:**
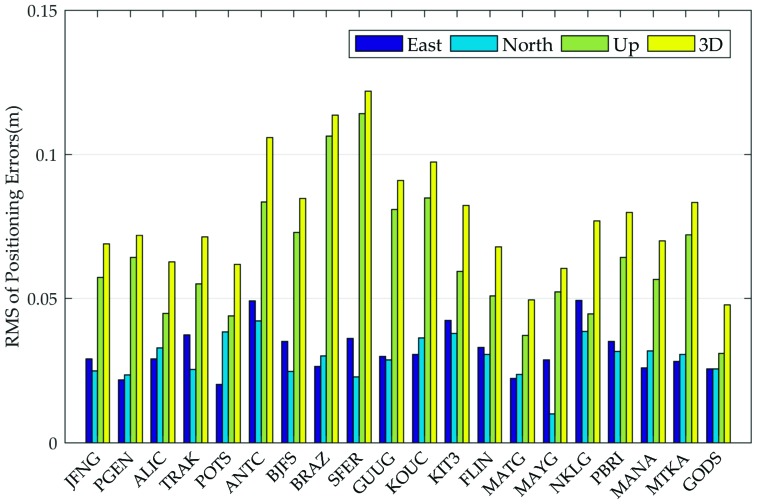
Daily RMS values of PPP positioning results at the 20 selected global IGS stations using MADOCA real-time products.

Subsequently, the daily RMS values of positioning errors in the east, north, up, and 3D directions for the 20 stations are calculated and depicted in Figure 12. It should be noted that the positioning results after 360 epochs (with a sampling rate of 30 s) were used for the daily RMS calculation to avoid the influence of convergence/initialization time on the positioning accuracy evaluation. After filter convergence, it is shown in Figure 12 that the real-time kinematic positioning accuracy using MADOCA real-time products is at centimeter level for horizontal direction and 1 decimeter for up direction, which may be attributed to MADOCA real-time 30-s high sampling rate orbit and clock products. The daily RMS values of all selected stations can reach 4.9, 4.2, 11.7, and 12.1 cm in the east, north, up, and 3D directions, respectively. This means that the current MADOCA real-time products can meet the accuracy requirements of the PPP solution and also demonstrate the validity and authenticity of the above-mentioned orbit and clock products evaluation work.

## 7. Summary and Conclusions

In this paper, a detailed description of QZSS and the MADOCA real-time messages was provided. The availability of MADOCA real-time products for three months was counted. The accuracy of orbit and clock of all available GPS satellites provided by MADOCA real-time products was assessed from both seven-day-short-term and one-year-long-run perspectives through comparison with IGS final products while PPP verification was also conducted from an application perspective.

The statistic results show that the epoch availability of MADOCA real-time products over three months for GPS satellites was about 92.6% and the vast majority of available epochs have over 30 usable GPS satellites. The seven-day epoch by epoch comparison results show that the orbits errors range from 1.8 cm to 3.9 cm after the unification of coordinate reference using the 7-parameters Helmert transformation model, and the clock errors range from 0.04 ns to 0.15 ns (1.2 cm to 4.5 cm in ranges). The corresponding orbit-error-only SISRE and SISRE for all available satellites are respectively less than 5 cm and 10 cm. In the long-term daily SISRE RMS statistics, the accuracy of MADOCA real-time products shows the consistent accuracy with the short-term epoch-by-epoch results except for a few jumps. The open source software RTKLIB was used on the daily kinematic PPP positioning evaluation, and over 20 selected IGS stations positioning results show 4.9, 4.2, 11.7 cm accuracy could be reached in the east, north, up directions after the convergence, respectively, which is consistent with the QZSS CLAS service.

In conclusion, the current MADOCA real-time ephemeris products prove to meet the accuracy demands of the RTPPP solution which will better serve the vast majority of RTPPP users who can receive the augmentation signals.
